# The missing indels: an estimate of indel variation in a human genome and analysis of factors that impede detection

**DOI:** 10.1093/nar/gkv677

**Published:** 2015-06-30

**Authors:** Yue Jiang, Andrei L. Turinsky, Michael Brudno

**Affiliations:** 1 Centre for Computational Medicine, Hospital for Sick Children, Toronto, ON, M5G 0A4, Canada; 2 Center for Biomedical Informatics, School of Computer Science and Technology, Harbin Institute of Technology, Harbin, Heilongjiang, 150001, China; 3 Program in Genetics and Genome Biology, Hospital for Sick Children, Toronto, ON, M5G 0A4, Canada; 4 Department of Computer Science, University of Toronto, Toronto, ON, M5S 3G4, Canada

## Abstract

With the development of High-Throughput Sequencing (HTS) thousands of human genomes have now been sequenced. Whenever different studies analyze the same genome they usually agree on the amount of single-nucleotide polymorphisms, but differ dramatically on the number of insertion and deletion variants (indels). Furthermore, there is evidence that indels are often severely under-reported. In this manuscript we derive the total number of indel variants in a human genome by combining data from different sequencing technologies, while assessing the indel detection accuracy. Our estimate of approximately 1 million indels in a Yoruban genome is much higher than the results reported in several recent HTS studies. We identify two key sources of difficulties in indel detection: the insufficient coverage, read length or alignment quality; and the presence of repeats, including short interspersed elements and homopolymers/dimers. We quantify the effect of these factors on indel detection. The quality of sequencing data plays a major role in improving indel detection by HTS methods. However, many indels exist in long homopolymers and repeats, where their detection is severely impeded. The true number of indel events is likely even higher than our current estimates, and new techniques and technologies will be required to detect them.

## INTRODUCTION

The introduction of High-Throughput Sequencing (HTS) technologies has enabled the sequencing of thousands of human genomes, both in large-scale population studies (1–3) and more recently in the efforts to understand the etiology of human disease (4–7). These studies commonly report the total amount of variation observed in the sequenced genomes, including the detected single nucleotide polymorphisms (SNPs) and insertions or deletions (indels). For example, the genome of J. Craig Venter (8), sequenced using the low-coverage Sanger method (9), was reported to contain 3 213 401 SNPs and 851 575 indels compared to the National Center for Biotechnology Information (NCBI) version 36 human genome reference assembly. The study of the genome of James Watson (10), sequenced with the 454 technology (11), reported 3.3 million SNPs and 222 718 indels. The more recent 1000 Genomes Consortium, mostly using the Illumina short read technology, reported 2 741 276 SNPs and 322 078 indels per high-coverage genome of European ancestry (average over three members of the same family), and 3 261 036 SNPs and 382 869 indels per Yoruban genome (12). More recently, we reported 592 373 indels in a European genome (NA12878) and 784 319 indels in a Yoruban genome (NA18507) (13). As these numbers illustrate, while estimates of the total amount of single nucleotide variation between a newly sequenced genome and the human reference are relatively consistent, the reported amount of insertion/deletion (indel) variation differs dramatically between studies. The latter is predominantly driven by small indels of length 1–20 bp, which make up 98.5% of all indel events (13). As has been previously noted, such major differences in indel variation are nearly certainly due more to the technical limitation of the various sequence-analysis technologies used in each study than to the actual genomic differences across the studied individuals (14).

### Identifying indels with Sanger sequencing

Sanger sequencing was the technology of choice for several decades enabling some of the most important early advances in genome sequencing. Among the main advantages of the Sanger method are longer sequencing reads and high reliability of the results (i.e. few false positives). However, the prohibitive cost of Sanger sequencing has led to a much lower genome coverage and throughput compared to HTS.

The first comprehensive study of indels in a human genome was performed on the genome of J. Craig Venter, which was sequenced with Sanger sequencing to the depth of 7.5x (8). The study detected 292 102 heterozygous and 559 473 homozygous indels in comparison to the NCBI reference genome, in addition to 3 213 401 SNPs or roughly 3.8 SNPs for every indel (S/I ratio). The authors also estimated that they missed 24.6% of all heterozygous variants due to allele dropout, leading to a corrected estimate of 387 403 heterozygous variants and 946 876 indels of both types. This result is concerning due to an extremely high fraction of homozygous indels. If all indels were common variants (with Minor Allele Frequency (MAF) ∼0.5) one would expect a roughly 2:1 heterozygous-to-homozygous ratio. Nor can such a large proportion of homozygous indels compared to heterozygous ones be explained by evolutionary selection. Rather it is likely to be an artifact, with either true heterozygous indels missed, homozygous indels falsely detected, or heterozygous indels mis-classified as homozygous (which would be possibly due to allele dropout).

Several studies have identified indels directly from Sanger traces. In these studies a total number of indels in a human genome is impossible to discern due to the low coverage of each individual genome. However, one can compute the S/I ratio, which is typically reported as ∼5:1 (3,14,15). Interestingly, an independent comparison of a portion of the *Homo sapiens* genome from Celera to the NCBI reference also resulted in an S/I ratio of 4.7:1 (16), somewhat higher than in the original analysis of Levy et al. (3.8:1).

### Identifying indels with HTS technologies

The recent popularization of HTS technologies (also referred to as next-generation sequencing) has led to the sequencing of hundreds of individual genomes. However, the number of indels reported by various studies ranges considerably: from 135 262 indels of size 1–3 bp reported in the Han Chinese genome (17), to ∼400 000 indels reported in a Yoruban genome NA18507 (18); for a comprehensive review, see (14). As already mentioned above, recent analysis from the 1000 Genomes Project yielded an average of 322 078 indels per individual in the European genome and 382 869 in the Yoruban genome (12). However, given the SNP counts reported in the study and assuming an S/I ratio of 4.7, one would expect 583 250 indels in a European genome and 693 837 indels in a Yoruban genome, which suggests that only ∼55% of all insertions and deletions were actually detected for each genome. Another recent study compared the observed number of indels with false-positive and false-negative rates estimated via simulations, and arrived at a whole-genome estimate of 665 684 indels in another Yoruban genome NA19240 (19). These estimates, however, are substantially smaller than 851 575 indels reported in (8) for Craig Venter's genome.

While evaluating PRISM (13) we analyzed two genomes sequenced with paired-end 100 bp Illumina reads, and identified 592 373 indels in the European individual NA12878 and 784 319 indels of size 1–100 bp in the Yoruban NA18507 genome. This contrasts with ∼400 000 indels reported in the same Yoruban genome by Bentley et al. (18). More surprisingly, even though our method showed ∼95% precision and recall on simulated data (and 90% accuracy via polymerase chain reaction validation), we were only able to identify 65% of the small indels (1–20 bp) previously characterized by Kidd et al. (2) via randomly sampled Sanger reads in NA12878. Correcting directly for the false negatives, we estimated ∼1.2×10^6^ indels in the Yoruban NA18507 genome, and ∼1×10^6^ indels in the European NA12878 genome—roughly in line with the estimates in (8), but significantly higher than in previous HTS-based studies.

### Challenges in indel detection

Various factors affecting the accuracy of indel detection have been previously investigated. Some of the challenges relate to the choice of the sequencing platform and strategies for the initial alignment of sequencing reads. For example, Illumina sequencing platform is noted for its suitability for indel detection due to its low indel error rate (20,21). The general difficulty of sequencing in repeat regions is also well known, especially in homopolymers (stretches of the same nucleotide). This difficulty is much more pronounced in the short-read HTS platforms, whereas Sanger sequencing is better able to unambiguously capture many of the short-repeat types in the genome, although at a high cost (20). Indels may also be missed if the alignment algorithm mishandles gap assignment, mistaking a true indel for a series of SNPs in a forced ungapped match. Therefore algorithms that perform gapped alignments in short reads effectively should be used for indel detection (21).

The concordance among different indel detection methods is generally low, which suggests that indel detection in human populations is likely to be rather incomplete (3,22,23). False negatives (i.e. missed indels) present a particular difficulty, as their frequency is difficult to directly validate, and therefore the estimation of the false negative rates across pipelines remains inadequate (22). Because the true indel landscape across the human genome remains unknown, simulated indel data sets are typically used to quantify the indel detection accuracy and its contributing factors, such as indel frequency, indel size, read length, read coverage, and software tool options (24–26). However, reliance on simulated data impedes an unbiased assessment of the relationship between the true indel distribution and the various intrinsic features of the genome, such as the presence of Alu and other repeats or the GC content. A promising new approach to detecting indels in repeat regions was recently developed for the whole exome sequencing (WES) data analysis (27). Using simulated and real WES data sets, a follow-up study quantified an enrichment of low-quality and/or discordant indels near homopolymers and short repeats, among other factors (28). However, the analysis was restricted to the regions around exons (± 20 bp), rather than genome-wide. Thus, despite a growing number of pipelines for indel detection, and a general consensus that many indels remain undetected by each of the pipeline, a reliable estimate of the number of the missing indels in a human genome as a whole has not yet been achieved.

In this study we present a novel method that estimates the genome-wide number of missing indels, and arrive at the total amount of indel variation present in a human genome. Our estimates of predictive accuracy are based on real rather than simulated genomic data. We use a combination of two sequencing technologies to derive these estimates, and test our approach on three indel callers: PRISM, GATK (29) and Dindel (30) while also using two sequence aligners, BWA (31) and BFAST (32). All pipelines yield consistent estimates of between 907 638 (based on GATK+BFAST results) and 972 159 (based on GATK+BWA results) insertion and deletion variants of length 1–10 bp in the NA18507 Yoruban genome. These estimates are more than twice the amount of variation reported by the 1000 Genomes project but are consistent with previously published Sanger-based studies such as (8). We investigate the reasons for the discrepancies in the identification of indels, focusing in particular on the limitations and biases of high throughput sequencing technologies, as well as the difficulty of indel detection in repeat elements and homopolymers. This allows us to highlight the main factors that lead to under-estimates of the total amount of indel variation in a human genome, especially at repetitive and low-complexity loci. We propose that in addition to factors such as sequencing read length and alignment quality, which have been known to affect indel detection, the length of homopolymers and other repeat regions, and the lack of *informative reads*, due to repeats, are among the key drivers for the ability to identify many indels genome-wide. We also investigate the effect of the GC content, as well as the two related issues of the sequencing coverage and the presence of Alu repeats, on the ability of HTS-based methods to identify indel events in whole genome sequencing data.

## MATERIALS AND METHODS

### Data-set summary

We have analyzed three sequencing data sets of the same Yoruban individual NA18507 from the 1000 Genomes project. These data sets include 36 bp paired-end Illumina reads (18) with 40x coverage, similar to the data used in the 1000 Genomes Pilot Project (12); longer 100 bp paired-end Illumina reads (NCBI SRA id: SRA010896) with 47x coverage; and Sanger sequencing traces (2) with 0.7x coverage.

The data on the Alu elements were retrieved from the *RepeatMasker* annotation of the human genome assembly hg18 (http://www.repeatmasker.org).

### Building an indel sequence for indel coverage calculation

For each detected insertion or deletion, we created a modified version of the genome that represents the mutation. This allowed us to investigate in detail the mapping of reads to both the modified version of the genome and/or the reference genome. The coverage and the alignment quality, as well as the observed proportion between the variant mappings and the reference mappings, were used in many subsequent computations.

**Figure 1.**
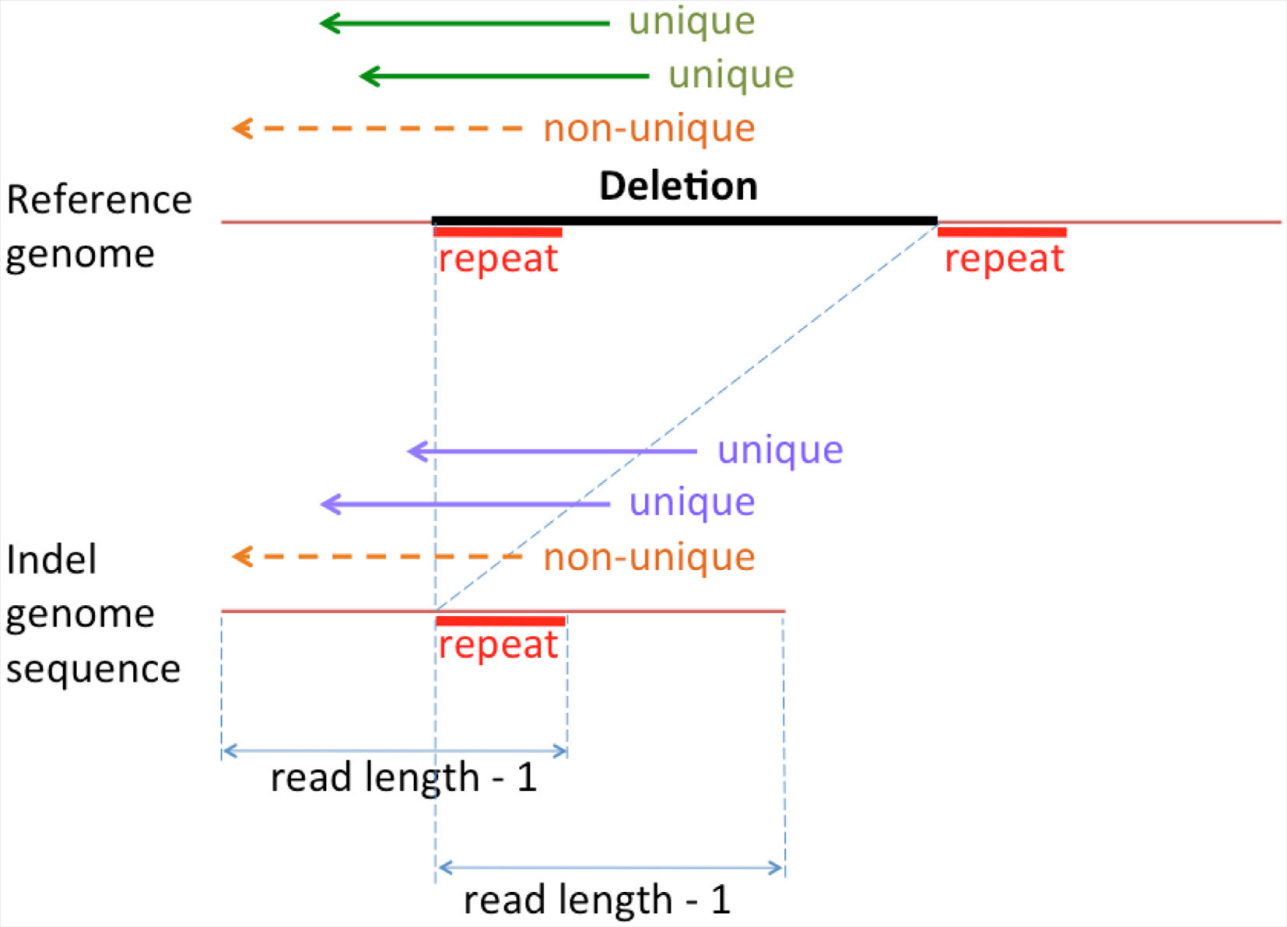
Modified reference genome and calculation of read coverage. Indel genome sequence for the deletion is created by gluing together the two flanking segments of the deletion, after which sequencing reads are aligned simultaneously to both reference and modified sequence. The potential presence of repeat sequences (marked in red) inside/outside the deletion may affect the uniqueness of read alignments: reads that span the entire repeat can be aligned uniquely to either the reference genome (green arrows) or the donor indel sequence (blue arrows), whereas reads not spanning the repeat region (orange dash arrows) will align to both reference and modified sequences equally well. The length of the flanking segments may differ due to read length and repeat length as shown. The coverage of the reference and alternate indel event is calculated as the number of reads that aligned uniquely to each (and not to both).

For each indel we built a small piece of sequence by gluing its flanking reference sequences with each other (for deletion) or with the inserted sequence (for insertion). The constructed sequence piece represents the derived individual genome at the indel position. The length of the constructed piece may differ depending on a selected read length, as well as on the length of the repeat sequence at the indel position in the reference genome. We retained only the reads of a given length that matched either the individual genome's sequence (when the individual genome contains the indel) or the corresponding reference genome region (when the individual genome has no indels) but not both equally well. We required all read alignments be without gaps, over the indel sequence itself and within the 10 flanking nucleotides on both sides of the indel. Figure 1 illustrates the process of building the new reference and the coverage calculation for each indel using only the uniquely aligned reads. This method is utilized for several analyses. The reads that uniquely align to either the indel or reference genome are used for indel coverage calculation and heterozygosity analysis.

### Indel detection

For all Illumina data sets, we first aligned the reads to the reference genome using BWA (31). Indels were then detected using PRISM (13) pipeline and post-processed using custom scripts. To validate our results, a similar procedure was performed by combining BWA (31) or BFAST (32) read aligners with GATK (29) or Dindel (30) indel callers (see Supplementary Materials for the parameters used). All human indels were based on the Human genome hg18 assembly, the assembly used for generating some of the earlier data sets.

The *sensitivity* of HTS pipelines such as PRISM was defined by comparing the indels identified from Illumina reads with the benchmark annotation set derived from Sanger sequencing. The *saturation* was defined as the proportion of PRISM indels that are present in the annotation set; this proportion is generally low given the substantial under-prediction of indels from low coverage Sanger sequencing.

### Heterozygosity analysis

Because PRISM results do not include heterozygosity annotation, we recovered this information for PRISM indels by utilizing the method introduced in the last section. For each PRISM indel we built the indel sequences based on the detected indels. We combined these indel sequences and the original reference genome to construct a new reference, and then realigned all 36 bp and 100 bp Illumina reads to it. For each indel we calculated the coverage of indel sequences and the coverage of corresponding original reference regions.

The heterozygosity of each indel was calculated by comparing the coverage. In the ideal case an indel should be considered homozygous when only the indel sequence has coverage, and heterozygous when both the indel sequence and corresponding original reference region have coverage. To improve reliability we considered only the indels with total coverage of 10 or more reads for heterozygosity analysis, where only uniquely aligned reads were counted. We called an indel *homozygous* when the indel-variant coverage was 3 or more reads (out of ≥10 total reads) and the reference coverage was either zero or one (i.e. at most one misaligned read allowed for each indel); and *heterozygous* when both the indel and the reference were covered by ≥ 3 reads each (out of ≥10 total reads). For the remaining indels the heterozygosity was marked as unknown. Sanger reads were aligned with BWA-SW (33). We called indels from every single Sanger read alignment whose mapping quality was at least 50.

The threshold of 3 reads of each kind (out of 10 or more reads) to establish heterozygosity is somewhat arbitrary, and a certain number of heterozygous alleles will be either misclassified as homozygous or unclassified. With this threshold, a low-coverage set of 10 uniquely aligned reads will have a 10.9% chance for the heterozygous indel to be either unclassified (9.8%) or misclassified as homozygous (1.1%) if assuming an equal binomial probability for a read to be drawn from either allele. As the coverage rises, the probability of misclassification diminishes rapidly: e.g. a set of 15 reads will have a mere 0.73% chance for the heterozygous indel to be either unclassified (0.67%) or misclassified as homozygous (0.05%).

### Estimating the number of indels in a human genome

The indel sets in NA18507 were generated from Illumina 100 bp reads and Sanger traces, and used to estimate the total indel number in NA18507. This process involves the estimation of several key values. First, we computed the false discovery rate of the HTS pipeline by using the portion of Illumina indels that have Sanger coverage. This allowed us to estimate the number of true positives in the full Illumina set. Conversely, the false discovery rate of the Sanger annotation set was computed by examining the Illumina support for Sanger indels. We then gauged the completeness of the Illumina read coverage of the Sanger indels. The latter two estimates allowed us to account for the false or unmatched Sanger indels, thereby re-calibrating the sensitivity of the HTS pipelines on the Sanger annotation set. Finally, the adjusted sensitivity of the pipeline was applied to the estimated number of true detected indels, which resulted in the total number of the indels in the NA18507 genome. The indel number estimations at various steps of this calculation are summarized in the Results sections, and the detailed explanations and the equations used are provided in the Supplementary Materials.

### Fraction of informative reads

We note that for each indel, only a fraction of its sequencing coverage can contribute to the detection of the variant. Consider a read that covers the indel but also aligns continuously to the reference genome, possibly at another location, which is usually caused by repeats. Such alignment will contain no signal, e.g. soft clipping, from which the indel-calling pipeline may infer the presence of an indel. We call such reads *uninformative* because they do not contribute to indel detection (although in a paired-read scenario some of them may align to the indel position correctly if properly anchored by a mate read). The remaining reads are *informative*, and contribute directly to the indel discovery.

For a given indel we defined its fraction of informative reads (FIR) as follows. We simulated all possible 100 bp long reads that start before and end after the indel, covering it entirely with at least 1 bp margin on both sides. For each deletion variant, there were 99 such indel-spanning reads, the first of which started at 99 bp upstream of the deletion and the last one started 1 bp right before the deletion. For an insertion variant, the number of the indel-spanning reads was 99 minus the insertion length. For each indel we aligned the simulated read set to the reference genome and identified those reads that did not align continuously, i.e. were informative. Their fraction among all reads simulated was defined as the FIR for that indel.

### Homopolymer annotation

To achieve better insight into the challenges of indel detection in repeat regions, we analyzed the indel distribution and detection in homopolymers and dimers. The *indel density* was defined as the number of indels per 1 Kbp or repeat region's length. The homopolymer/dimer length used in this paper is the length of the region in the reference genome. For each position in the reference genome we consider a repeat unit of length 1 and 2 bp (i.e. homopolymer and dimer) starting from there. We then check how many repeat units are following. We finally annotate current position's repeat type as the one with most repeat units, and start over from the position right after the last unit. Indels were annotated by the longest repeat that they overlap. For dimers the repeat length is the number of repeated nucleic acid pairs (e.g. a four-nucleotide sequence ACAC has the repeat length 2; dimers of length 1 are not considered because they do not contain repeats).

### Comparison of indels across species

We analyzed the indel distributions in four primate genomes: Chimpanzee, Gorilla, Orangutan and Rhesus Macaque, comparing their genomes to the Human reference genome. Indels for the primate species were detected with respect to the Human genome assembly hg19, for which we used multiple alignments of 45 vertebrate genomes provided by the UCSC Genome Browser (34), available at http://hgdownload.soe.ucsc.edu/goldenPath/hg19/multiz46way/. For the purpose of comparison with primate indels, the human indels in the NA18507 genome were converted to the hg19 genomic coordinates using the UCSC *liftOver* software utility (34). To facilitate the cross-species comparisons, we normalized the counts of indels of each length by the number of 1 bp indels.

## RESULTS

In the following sections, we first quantify the sensitivity of indel detection by HTS methods, compared to indels previously identified by Sanger sequencing, and analyze various sequence features that affect sensitivity. Subsequently, we combine the sensitivity analysis with an estimate of the false discovery rate to calculate the approximate number of indels in a full human genome. To better explain the discrepancy between our results and previous HTS-based studies we concentrate on indels in homopolymer regions, which present the most difficulty for shorter HTS reads. Our results are consistent with the earlier observations that the indel rate in such regions is significantly higher, and grows rapidly with homopolymer length, while at the same time error rates due to technological and biological artifacts also increase and eventually become dominant, impeding the detection of indels (30).

### Analysis of indel detection ability

Our initial goal is to quantify the sensitivity of indel detection using HTS data. Table 1 compares PRISM indels identified from Illumina 100 bp and 36 bp reads with the results of (2), using the Kidd set (226 112 indels) as the benchmark. As expected, the saturation (see Materials and Methods) is low due to the low coverage of Sanger sequencing. The sensitivity of detecting indels from the 100 bp read data set was much higher than from 36 bp reads (64.5% versus 35.5%), and only 0.9% of Kidd set indels were supported exclusively by 36 bp reads. However, as many as 80 168 indels (35.5%) in the Kidd benchmark set, derived from Sanger sequencing, were not detected by PRISM even using the longer 100 bp reads. To understand the reasons for such a large number of false negatives, first we used the Kidd indel data set to build a modified reference genome that includes both the original reference and the alternate indel events, as described in Materials and Methods (Figure 1). We then aligned the 100 bp reads to this new reference, and considered the number of reads uniquely aligned to each indel sequence as its supporting evidence.

**Table 1.** Comparison of indel detection in PRISM and in (2)

Read Data	Indels in PRISM	Indels in Kidd et al.	Sensitivity	Saturation	Fold increase
100 bp	784 319	145 944	64.5%	18.6%	5.4
36 bp	382 257	80 163	35.5%	21.0%	4.8
100 bp ∩ 36 bp	353 696	78 017	34.5%	22.1%	4.5
100 bp exclusive	430 623	67 927	30.0%	15.8%	6.3
36 bp exclusive	28 561	2146	0.9%	7.5%	13.3

The number of indels detected by PRISM using read data of different length is shown along with the number of indels recovered in each case from the Kidd set. Sensitivity is the fraction of all 226 112 indels from the Kidd set detected by PRISM using different read libraries. Saturation is the fraction of PRISM indels that are in the Kidd set, and fold increase is the ratio between the sizes of PRISM and Kidd result sets (i.e. reciprocal of the saturation). Both saturation and fold increase demonstrate the relative abundance of PRISM indels compared to the Sanger-based results of (2).

**Figure 2.**
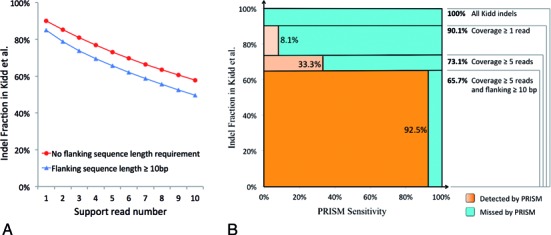
Effect of sequence coverage and alignment quality on indel detection. (**A**) Fraction of indels from Kidd et al. set with different support read numbers. ‘No flanking sequence length requirement’ (red curve) shows the fraction of indels in Kidd et al. that are supported by different number of Illumina 100 bp reads. ‘Flanking length at least 10 bp’ (blue curve) additionally requires the support reads to have at least 10 bp sequence on both sides of the breakpoints of indels. (**B**) Separation of the Kidd indel set into four categories based on the read coverage and alignment quality: no read coverage, coverage by 1–4 read, coverage by at least 5 reads, and coverage by at least 5 reads with additional flanking sequence alignment of a least 10 bp. The *X*-axis represents the PRISM detection sensitivity for each category of Kidd indels. The *Y*-axis is proportional to the size of each category. The area of each box represents the relative proportion of the indels either detected (orange) or missed (blue) by PRISM. The sensitivity of PRISM detection is defined by the combined orange fractions of the square. The vast majority of the detected Kidd indels are those with high coverage and high-quality alignment (dark-orange box).

Surprisingly, 90.1% of indels in the overall Kidd benchmark set were supported by at least 1 read, and 73.1% were supported by at least 5 reads, which was the read count threshold used in PRISM (Figure 2A). Most of the indels that were supported by ≥ 5 reads were detected by PRISM (sensitivity 86.4%), and those that were missed had low sequencing quality and/or alignment issues with the corresponding reads.

We then additionally required the sequence alignment of ≥10 bp on both sides of the indel breakpoint (which is also required by PRISM). Only 65.7% of the Kidd indels had the support of ≥ 5 reads with the required alignment stringency (Figure 2A). The PRISM sensitivity on this set was 94.6%, which suggests that the overall PRISM sensitivity rate of 64.5% is defined almost entirely by this high-quality subset of indels. Only about 6% of the detected indels failed to meet these criteria (Figure 2B, light-orange portions). Conversely, we characterized the causes of failure to detect Kidd indels as follows: 72% of false negatives were missed due to absent or insufficient sequencing coverage, 14% were missed due to alignment issues, while the remaining 14% were missed for various other reasons (Figure 2B, blue portions). It is worth noting that sequencing coverage and alignment quality are related issues: e.g. artifacts causing poor read alignment would also lead to more rejected reads and would thus reduce the coverage.

### GC content and Alu elements

**Figure 3.**
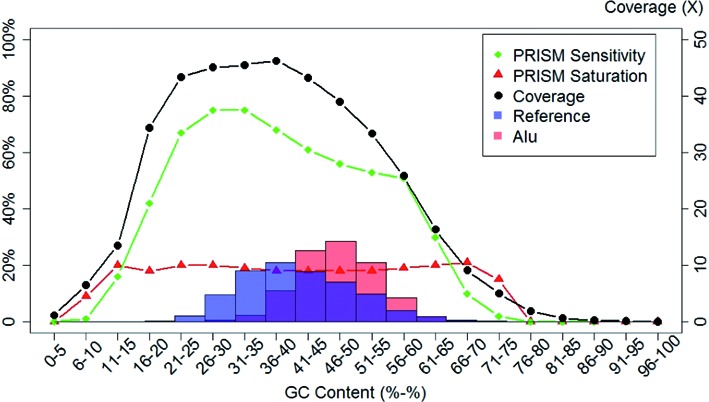
Dependence of coverage and PRISM indel-detection metrics on the GC content. The reference genome was cut into 200 bp pieces and binned by GC content. PRISM indel detection sensitivity (green curve) and saturation (red curve) are shown for each bin along with the genome coverage (black curve). Also shown is the distribution of the full reference genome across the same GC bins (blue semi-transparent histogram), as well as the distribution of Alu elements (red semi-transparent histogram). The two histograms demonstrate that Alus are generally overrepresented in areas with higher GC content, and also that the noticeable dip in the PRISM sensitivity corresponds well with the presence of Alu elements (pink and magenta areas of the Alu histogram).

Since Illumina sequencing has coverage biases related to GC composition (35), we further investigated the ability to detect indels on genomic regions with different GC content. Figure 3 illustrates the fraction of the genome, coverage, and PRISM sensitivity and saturation in each of the GC content bins. The results show that the Illumina sequencing coverage has an obvious bias towards regions with 20–55% GC content, and that PRISM's indel detection ability is positively and strongly correlated with coverage. PRISM's saturation becomes stable when coverage reaches ∼10x, perhaps reflecting the true relationship between the detection abilities of PRISM versus Sanger-based method of (2), where PRISM has roughly 5-fold increase in the power of indel detection (as evidenced also in Table 1). Meanwhile PRISM sensitivity keeps increasing along with the coverage.

Regions with GC content of 26–40% attract the highest coverage and also give rise to the best PRISM performance. However, although the coverage across these GC bins is very similar (between 45.1 and 46.3), the sensitivity of PRISM drops from 74.6% to 67.6% after the GC content exceeds 35%. To identify the cause of this decrease in sensitivity, we analyzed four separate groups of indels: indels supported by ≥ 10 reads, indels at least 5 Mbp away from telomeres and centromeres, indels outside segmental duplications, and indels outside of annotated Alu repeats (Supplementary Figure S1). The performance drop is consistent in all of these analyses except relative to Alu repeats. In non-Alu regions the sensitivity decreased by only 1.1%, from 78.8% to 76.7 between the 31–35% and 36–40% GC bins. Meanwhile the saturation metrics in the two bins were very comparable at 20% and 19.6%, respectively. In contrast, for indels within Alu repeats, the sensitivity was only 47%, which was much lower than the genome-wide non-Alu average of 73%. The saturation in Alus was 16%, which was also lower than the 20% saturation rate outside of Alu repeats.

We then plotted Alu distribution across GC bins (Figure 3). The histogram showed that Alu percentage is highest between 36–40% and 56–60% GC bins, consistent with regions of the genome where indel detection sensitivity drops. Given the well-known enrichment of Alu elements in GC-rich genomic regions, this may explain why PRISM sensitivity declines in higher GC content bins. Similar results were observed using GATK pipeline (Supplementary Figure S2). More generally, these results suggest that the presence of Alu repeat regions can have a major effect on the ability of PRISM and similar pipelines to detect indels.

### Estimation of indel numbers in the Yoruban genome NA18507

**Figure 4.**
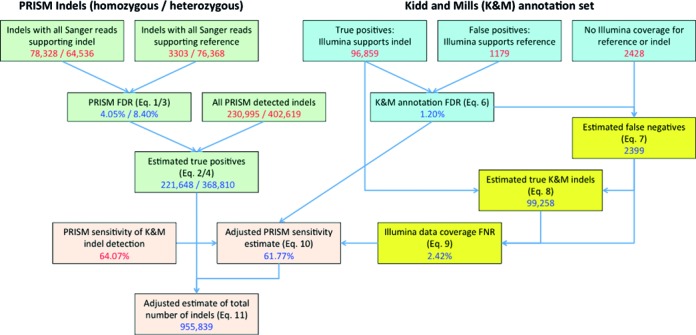
Estimation of the total number of 1–10 bp indels in the Yoruban genome NA18507 using PRISM in combination with BWA read aligner. The workflow involves the estimation of four sets of values: PRISM FDR and the number of true positive indels detected by PRISM (green boxes); the reliability of the reference indel annotation combined from Kidd and Mills sets (via false discovery rate, FDR; blue boxes); the incompleteness of the Illumina read coverage of the reference indels (via false negative rate, FNR; yellow boxes); and the computation of the adjusted sensitivity of indel detection in PRISM, which is used to estimate the overall number of indels in the genome (orange boxes). Each box shows the initial indel counts or pipeline sensitivity (red numbers) as well as the computed estimates (blue numbers) based on the equations indicated in parentheses. The detailed explanation of the equations and the workflow is presented in the Supplementary Materials.

To estimate the total number of indels in a single human genome, we jointly analyzed the Illumina 100 bp and Sanger sequencing data from the genome. This process is summarized in Figure 4 with additional details provided in the Supplementary Materials. Our focus was indels of length 1–10 bp, which can be detected most confidently, and comprise the vast majority of all indel variation. To maximize the confidence of Sanger sequencing-based indels, we selected indels reported in both (2) and (3) as our benchmark annotation data set, which includes 123 967 indels, of which 120 056 had length 1–10 bp.

#### False discovery rate of PRISM

First we estimated the false discovery rate (FDR) of PRISM indel detection on Illumina 100 bp reads, defined as the fraction of indels identified in Illumina data that are rejected by Sanger data. This was done separately for homozygous and heterozygous variants (Figure 4, green boxes, see equations (1)–(4) in the Supplementary Materials). 633 614 of PRISM indels had coverage of at least 10 reads. For 230 995 of them all reads supported the alternate allele (homozygous) and the remaining 402 619 had reads supporting both the alternate and the reference (heterozygous). We estimated the FDR on the subset of homozygous indels that had either a clear support or a clear rejection by all covering Sanger reads. This subset comprised 78 328 homozygous indels that were supported by all covering Sanger reads (true positives), and 3303 indels that had all covering Sanger reads supporting the reference genome (false positives). According to equations (1) and (2) the FDR of homozygous indel detection was 4.05%, suggesting that 95.95% of all detected homozygous indels were true discoveries. This gives the adjusted estimate of 221 648 true-positive homozygous indels in the NA18507 genome. Similarly, 64 536 heterozygous indels were supported by all covering Sanger reads, while 76 368 had all covering Sanger reads supporting the reference genome. Hence according to equations (3) and (4) the FDR of heterozygous indel detection was 8.40%, giving the adjusted estimate of 368 810 true-positive heterozygous indels in the NA18507 genome.

#### False discovery rate of Sanger annotation

We estimated the accuracy of the Sanger-based indels (referred to as annotation) by examining their coverage by the Illumina 100 bp reads (Figure 4, blue boxes, equation (6)). The Illumina reads were aligned to a modified reference genome representing all Sanger-based indels. Of the 120 056 indels, *n* = 98 038 had coverage by at least 10 Illumina reads (for either allele). We used this high-coverage subset to estimate the FDR in the combined Kidd and Mills data. 1179 of the high-coverage indels had no Illumina reads supporting the indel variant and were thus considered false positives; the remaining 96 859 indels were considered true positives. According to equation (6) the FDR_ann_ = 1179/98 038 = 1.20%.

#### False negative rate of Illumina coverage

Next we accounted for the rate at which indels may be missed due to lack of Illumina coverage. The false negative rate (FNR) is defined as the fraction of true Sanger indels that had no Illumina support. There were 2428 indels in the Kidd and Mills annotation data set that had no coverage (of either reference or alternate alleles) by Illumina 100 bp reads. Adjusting for the annotation's FDR, equation (7) suggests that 98.8% of them, or 2399 indels, were probably true Sanger indels (false negatives). This increases the estimated number of true Sanger indels to 96 859 + 2399 = 99 258 (equation (8)) of which 2399 have no Illumina support, yielding FNR_data_ = 2399/99 258 = 2.42% (equation (9)).

#### Annotation of indel zygosity

The analysis of Sanger indel zygosity required sufficient coverage by Illumina reads (see Materials and Methods). It was therefore performed on the subset of 96 859 Sanger indels that had high support by Illumina data. Of these 44 345 (or 46%) were homozygous indels supported by all the covering Illumina reads, while 52 514 (or 54%) were heterozygous indels covered by Illumina reads matching both the indel variant and the reference genome. However, the expected ratios of homo- and heterozygous indels are 33% and 67% respectively. This suggested that although the annotation combined from (2) and (3) is highly reliable (FDR = 1.2%), it is (unsurprisingly) biased towards homozygous indels, as at very low coverage many heterozygous indels are not sampled.

#### Total estimate

Finally we estimated the total number of indels in the NA18507 genome (Figure 4, pink boxes). The sensitivity of PRISM indel detection on the combined Sanger indel annotation set was 64.07%, which was further adjusted to 61.77% according to equation (10) after taking into account the FDR of the Sanger annotation and the FNR of the Illumina coverage. Using this adjusted PRISM sensitivity leads to the estimated total of 955 839 indels in the genome according to equation (11).

We also analyzed separately the indels located in non-homopolymers and in short homopolymers of size up to 10 bp by following a similar process (Supplementary Table S1). This led to an estimate of 639 077 indels i.e. 66.9% of the total indel estimate. These results suggest that long homopolymers contain roughly one-third of all estimated indels. Furthermore, we can expect a considerable fraction of indels in dimers and other microsatellite repeats.

### Validation of the indel estimate

We validated our results by using other pipeline tools for a similar analysis of indels of length 1–10 bp. Two different sequence aligners, BWA (31) and BFAST (32), were used in combination with two different indel callers, GATK (29) and Dindel (30). The read mappers managed to align a comparable number of reads: 1 165 635 168 for BWA and 974 469 078 for BFAST. The four combinations of tools return different numbers of the indels, ranging rather widely from 555 629 (for GATK+BFAST) to 803 633 (for Dindel+BWA) detected indels, with an overlap of 369 641 indels across all pipelines (Supplementary Section 3 and Figure S3). Despite these differences, there was a good agreement on the total number of indels in NA18507 after our estimation procedure was applied to the output of each pipeline. The estimates ranged from 907 638 (for GATK+BFAST) to 972 174 (for GATK+BWA)—numbers very similar to the 955 839 estimate derived above from PRISM results (Supplementary Figures S4–S7). Similarly, analysis of indels located in non-homopolymers and in short homopolymers of size up to 10 bp was consistent with the PRISM results: such regions contained between 63.4% and 67.1% of all estimated indels in the genome, confirming the earlier indication that long homopolymers contain about one-third of all estimated indels (Supplementary Table S1).

### Coverage depth and quality of reads for missing indels

**Figure 5.**
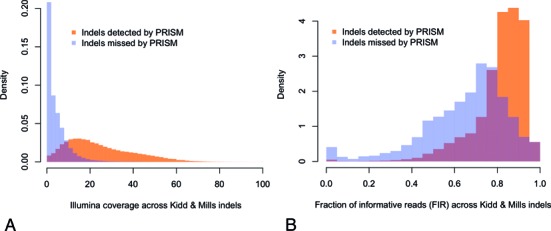
Coverage and FIR for the Kidd and Mills indel annotation set. (**A**) The distribution of the number of covering Illumina reads is shown separately for the indels detected by PRISM (orange) and for the missed indels (blue). The overlap between the two histograms is shown in brown. To compensate for the unequal number of detected and missed indels, each histogram is normalized to have the area = 1, i.e. representing the probability density. Only the coverage range of up to 100X is shown. Most of the detected indels are covered by at least 10 reads whereas most missed indels have coverage <10 reads. (**B**) The normalized histograms of the FIR are shown separately for the Kidd & Mills indels detected by PRISM (orange) and for the missed indels (blue), with the overlap shown in brown. Only the indels with at least 10 covering reads are used for the analysis. The detected indels tend to have a higher FIR.

We separated the Kidd and Mills annotation data set into those detected by PRISM and those missed by PRISM, and examined their coverage by Illumina reads. Higher read coverage appears to be strongly associated with higher rate of indel detection (Figure 5A). Among the indels detected by PRISM the median coverage was 22 reads, with 88% of the detected indels covered by at least 10 reads. In contrast, the median coverage across the missed indels was only 4 reads, and 83% of them were covered by less than 10 reads.

A further technical difficulty in the detection of indels is the low FIR (see Materials and Methods). The presence of repeat regions is the main scenario in which there is a significant drop in the FIR: typically uninformative reads are those that match another, unperturbed copy of a repeat elsewhere in the genome.

To quantify this factor we examined the FIR for indels from the Kidd and Mills annotation data set. We focused on the ability of PRISM to detect well-covered indels (with ≥10 Illumina reads), thereby excluding the possibility that any indels were missed due to low Illumina coverage *per se*. The results show that higher indel detection ability was clearly associated with a higher FIR (Figure 5B). The detected indels had a mean FIR of 81.3, compared to only 65.5 for missed indels (Wilcoxon rank sum test *P*-value < 2.2e-16). Only 2.7% of detected indels had an FIR < 0.5, compared to 18.8% of missed indels. A low FIR indicates that even if the indel coverage seems adequate, the *effective coverage*—i.e. coverage by informative reads—may be too low for the detection of the indel. This suggests that the difference in effective coverage between the detected and missed indels is even more pronounced than in Figure 5A: not only do missing indels have much lower coverage overall but also a smaller fraction of those covering reads contribute to the indel detection.

### Indel detection in homopolymer and dimer regions

**Figure 6.**
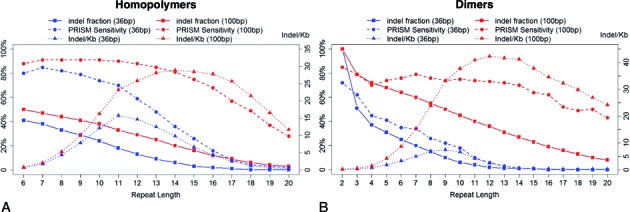
Quantification of indel detection by PRISM in homopolymers (A) and dimers (B). ‘36 bp’ and ‘100 bp’ stand for 36 bp and 100 bp read sets respectively. (**A**) The PRISM detection sensitivity is defined using the reference set from (Kidd et al. 2008). The indel density is the number of detected indels per 1 Kbp of the homopolymer region. The residual indel fraction for a given length is the proportion of the detected indels in homopolymers of the same length or longer (i.e. the reverse of the cumulative distribution function). As the length of homopolymers grows, the indel density keeps growing at first, suggesting that more indels exist in longer homopolymers. After a peak the indel densities start to decrease along with the PRISM sensitivity, suggesting that the density is affected by the difficulty of detecting indels in longer homopolymers. (**B**) Similar metrics were computed for indels detected in dimer regions, and similar patterns were observed.

The challenges of indel detection in repeat regions are further illustrated in Figure 6, which shows the distribution of PRISM indel-detection sensitivity and several other metrics in homopolymers (length > 5 bp) and dimers, using either the 36 bp or 100 bp reads. The indel density exhibits two different modes of behavior. As an illustration, consider the results derived from the 100 bp read set in homopolymers (Figure 6A, red curves). At first, as the homopolymer regions become longer, the indel density grows rapidly while PRISM detection sensitivity remains stable. However, after the homopolymer length exceeds 11 bp, the opposite tendency becomes evident: the density growth is suppressed so that the density quickly reaches its peak for homopolymers of size 14 bp and then declines dramatically, while the sensitivity of indel detection also starts dropping sharply.

A similar behavior is evident in all other scenarios, with the decline in indel density and indel-detection sensitivity much more pronounced for 36 bp reads than for 100 bp reads. This suggests that the decrease in the indel density in longer repeats is probably due to technical artifacts and the limitations of our indel-detection methods, and a similar observation was made in (30). Indeed, we believe that there is potentially a much larger number of indels in long repeats, which cannot be detected by current sequencing technologies and analysis methods. It follows that our calculations from the previous section, which indicated that roughly one-third of all indels are located in homopolymers longer than 10 bp, are likely to be a severe underestimate of the true indel mutation rate in repeat regions.

### Evolutionary analysis of indel accumulation in human and other primates

We propose that although our procedure for estimating the indel variation reflects the *apparent* number of indels in the genome, it is still lower than the true number of indel events that have occurred during the genome's evolution. A further factor that leads to underestimation of true indel rates at low complexity loci may be an evolutionary artifact, where multiple small insertions and deletions (of the same nucleotides) occurring in different parts of the homopolymer over time, are no longer distinguishable. As an example, consider two different 1 bp deletions occurring at the opposite ends of a homopolymer of length 10 bp. In this case sequence analysis methods cannot identify the original indel events directly because only their accumulated effect is observed—namely, the overall reduction of the homopolymer by 2 bp. The best an indel detection pipeline can do is to detect a single 2 bp deletion. In contrast, two short deletions occurring at 10 bp apart outside of homopolymers will leave two separate marks on the genome and should therefore remain detectable in principle.

We tested the evidence for this hypothesis by taking the human genome as a frame of reference and considering the genomes of several primate species. The larger the evolutionary distance between species, the more individual insertions and deletions should have occurred throughout a large repeat regions, compounding each other's effects; and hence the more of the resulting ‘cumulative’ indels should appear. Therefore the species that are closer to human (e.g. chimpanzee) should have a relatively lower fraction of long indels in homopolymer regions than the more evolutionarily distant species (e.g. macaque).

**Figure 7.**
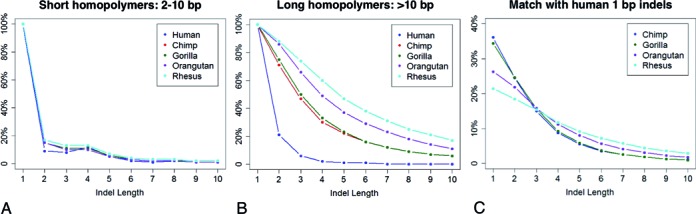
Comparison of indel length distribution across homopolymers in human and primate species. (**A**) Length distribution of indels located in short homopolymers (2–10 bp) in human and four primate species. The number of 1–10 bp indels was normalized to the number of 1 bp indels. The species are listed in an ascending order of the distance to human in the evolutionary tree, i.e. human, chimpanzee, gorilla, orangutan and macaque. The fractions of indels of different length are consistent among the five species. (**B**) Normalized length distribution of indels in homopolymers over 10 bp long in human and four primates. The proportions of long indels have positive relation to the evolutionary distance between the human and primate. (**C**) Distribution of indel lengths in four primate species in homopolymers 10 bp or longer where human has 1 bp indel. The farther a primate species is located from human, the more biased the corresponding distribution is towards longer indels.

To validate this assumption we used the indel set detected in our previous analysis as a sample of human genome, and compared it to the indels of four different primate species: Chimpanzee, Gorilla, Orangutan and Rhesus Macaque (listed here in the order of increasing evolutionary distance to Human) as described in Materials and Methods. The resulting indel-length distributions are shown in Figure 7. The fractions of longer indels corresponding to non-homopolymer and short (2–10 bp) homopolymer regions are very stable across all the five species (Figure 7A and Supplementary Figure S8). However, in longer (>10 bp) homopolymers, the proportion of longer indels correlates positively with the evolutionary distance of the primate species to human (Figure 7B), supporting our hypothesis that indels in homopolymers accumulate and merge during the evolution. Further evidence is presented in Figure 7C, which shows the indel length distributions of the four primate species, considering only the locations where analysis of NA18507 identified 1 bp indels. Species at a larger evolutionary distance from human have indel length distributions biased more towards longer indels, which is consistent with the pattern in Figure 7B.

## DISCUSSION

With the development of HTS over one thousand human genomes have been sequenced and several of them have been thoroughly analyzed. Although different studies usually agree on the amount of single-nucleotide variation in a genome, the estimates of indel variation across studies have differed dramatically. In the present manuscript we have analyzed the strengths and shortcomings of HTS-based technologies for identifying indels in personal human genomes. We used our analysis to estimate the total number of indel polymorphisms in a human genome, arriving at the estimate of ∼1 million indels in a Yoruban genome, consistent with earlier Sanger-based studies (8), but significantly higher than recent HTS-based analyses (10,12). Interestingly, applying the S/I ratio of 4.7 reported for the Celera genome in (16) to the SNP counts from (8) would result in the estimated 683 702 indels for Craig Venter's genome; whereas (8) reports 851 575 indels found directly from Sanger data, corresponding to an S/I ratio of 3.8. However, both of these numbers are still lower than our adjusted total estimates for the Yoruban genome, derived from PRISM, GATK and Dindel results with different read aligners. Using data from two complementary sequencing platforms, longer but low-coverage Sanger reads and shorter but higher-coverage Illumina reads, was key to revealing the much larger extent of indel variation in the genome; whereas estimates based on only one of these technologies result in significant biases and potential under-reporting of the total amount of variation.

We also demonstrate that the presence of repeat regions such as homopolymers, dimers and Alu elements may account for a disproportionately large number of undetected indels. For example, our results show that at least one-third of all indels occur in long homopolymers (>10 bp), which are the regions with high rates of sequencing errors and known difficulties for indel analysis (28,30). It appears that the negative effect of longer repeats on indel detection may not be easily mitigated by improving the sequencing quality or coverage, but is of a more fundamental nature.

We also present evidence that under-reporting of indels in longer homopolymers is exacerbated by an evolutionary process, whereby the effects of multiple individual indels are merged and can no longer be distinguished. Our results support the hypothesis that indel mutations actually occur at a higher rate than what can be discerned from the sequence alignment. We present evidence that evolutionary distance does not seem to affect the indel length in non-homopolymers and short homopolymers. On the other hand, it appears that in longer homopolymers, larger evolutionary time period (e.g. between the common ancestor of human and a primate) is associated with an overrepresentation of large indels, potentially concealing the true rate of indel variation in the genome.

## Supplementary Material

SUPPLEMENTARY DATA
