# Supplementary material for: The missing indels: an estimate of indel variation in a human genome and analysis of factors that impede detection
Source: Nucleic Acids Res. 2015 Jun 30;43(15):7217–28. doi: 10.1093/nar/gkv677 (PMC4551921; doi:10.1093/nar/gkv677)
Supplement: SUPPLEMENTARY DATA [file supp_43_15_7217__index.html]

The missing indels: an estimate of indel variation in a human genome and analysis of factors that impede detection — SUPPLEMENTARY DATA 

# The missing indels: an estimate of indel variation in a human genome and analysis of factors that impede detection

## SUPPLEMENTARY DATA

- SUPPLEMENTARY DATA
