# Supplementary material for: The missing indels: an estimate of indel variation in a human genome and analysis of factors that impede detection
Source: Nucleic Acids Res. 2015 Jun 30;43(15):7217–28. doi: 10.1093/nar/gkv677 (PMC4551921; doi:10.1093/nar/gkv677)
Supplement: SUPPLEMENTARY DATA [file supp_gkv677_nar-00676-z-2015-File009.pdf]

The missing indels: an estimate of indel variation in a human genome and analysis of factors that impede detection

Supplementary materials

Section 1: Estimating the number of indels in a human genome

The indel sets in NA18507 were generated from Illumina 100bp reads and Sanger traces, and used to estimate the total indel number in NA18507. We first used Sanger traces to validate Illumina indels. Our calculation was based on counting Illumina indels with Sanger coverage (i.e. covered by a Sanger read). For each Sanger read which covers an Illumina indel there are three situations: a) the Sanger read supports the indel i.e. either has exactly the same indel or has a indel that can be shifted to the Illumina indel by introducing at most one mismatch in the alignment; b) the Sanger read rejects the indel i.e. has a continuous segment covering the Illumina indel with at least 5bp flanking sequences on both sides of the indel; c) the Sanger read supports a different indel i.e. has a different indel within 5bp of the indel which cannot be shifted as in a).

We refer to a reported Illumina homozygous indel as *true positive* if all covering Sanger reads support it (situation a) and as *false positive* if all covering Sanger reads reject it (situation b). Define *hom* as total detected homozygous indels, hom_a and hom_b as the number of homozygous indels detected in Illumina reads with all covering Sanger reads supporting or rejecting the indel, respectively. Then the false discovery rate of homozygous indel detection is defined as the proportion of the rejected indels among all detected homozygous indels with high Sanger coverage:

$$FDR_{hom} = hom_b/(hom_a+hom_b). \qquad (1)$$

The number of true positive homozygous indels is estimated as

$$TP_{hom} = hom * (1 - FDR_{hom}). \qquad (2)$$

For heterozygous indels the Sanger reads should ideally come from both the variant allele (supporting the indel) and the reference allele (rejecting the indel). However, due to the very low Sanger coverage, requiring that both alleles be supported by Sanger data is too stringent for the majority of heterozygous indels. Instead we chose a pragmatic approach of gauging the FDR by examining the imbalance between the indel coverage and the reference coverage. Specifically, we focused on the numbers of heterozygous indels with all covering Sanger reads supporting/rejecting the indel. Define *het* as total detected heterozygous indels, and het_a and het_b as the numbers of heterozygous indels detected in Illumina data with all covering Sanger reads supporting/rejecting the indel, respectively. For true positive heterozygous indels the probability of full support and full rejection is 50:50 and so het_a and het_b should be close to each other. Therefore the difference between het_b and het_a can be used to estimate FDR for heterozygous indel detection as follows:

$$FDR_{het} = |het_b - het_a|/(het_a+het_b). \qquad (3)$$

The number of true positive heterozygous indels is estimated as

$$TP_{het} = het*(1 - FDR_{het}). \qquad (4)$$

Then we estimated the sensitivity of indel detection, i.e. the fraction of true indels from the reference annotation set that were detected correctly. Since most annotations lack heterozygosity information we compared homozygous and heterozygous indels to the same annotation and used the same sensitivity estimate for both indel types. Define *s* as the sensitivity of indel detection on an annotation. Then the preliminary estimate for the total indel number is estimated as

$$N = (TP_{hom} + TP_{het}) / s = (hom * (1 - FDR_{hom}) + het * (1 - FDR_{het})) / s. \quad (5)$$

However, this estimate does not yet take into account two additional sources of indel loss. First, although most of the existing indel annotations are validated, they may still contain false positives. Second, due to different sequencing technologies and analysis methods certain read sets may not be able to detect some of the real indels in an annotation.

To evaluate the FDR of a Sanger-based indel annotation we built a new reference with the indel sequences and reference genome, as described in the *Materials and Methods* section of the main text, and aligned high coverage Illumina sequencing data to it. Indels with coverage but no supporting reads for the indel variant can be considered false positives. Define *fp* as the number of these false positive indels and *n* as the number of all indels with Illumina reads. Then the false discovery rate of the annotation is

$$FDR_{ann} = fp/n. \quad (6)$$

Next, to quantify the incompleteness of Illumina coverage, define *no_cov* as the number of indels without Illumina coverage. Depending on the previously estimated FDR_{ann}, a fraction of them may not be true indels, but the remaining ones should be considered true indels that were missed due to incomplete Illumina coverage (i.e. false negatives). The number of such false negatives, *fn*, is estimates as

$$fn = no\_cov * (1 - FDR_{ann}). \quad (7)$$

This suggests that true Sanger-annotation indels comprise *(n-fp)* indels that have Illumina coverage (true positives), and *fn* indels that were missed by Illumina reads (false negatives):

$$true\_ann = n – fp + fn \quad (8)$$

Therefore the false negative rate of the Illumina sequencing data is defines as

$$FNR_{data} = \text{fn} / true\_ann = fn / (n – fp + fn). \quad (9)$$

Using the false discovery rate of the annotation, and adjusting further for the incompleteness of Illumina sequencing coverage, the adjusted sensitivity *s'* is defined as:

$$s' = s * (1 - FDR_{ann}) * (1 – FNR_{data}) \quad (10)$$

The preliminary estimate in equation (5) is then replaced with the adjusted estimate of the total number of indels:

$$N_{adjusted} = (TP_{hom} + TP_{het}) / s'. \quad (11)$$

Section 2: Analysis tools and parameters

BWA (v0.6.1) was used to align the Illumina reads and BWA-SW (v0.6.1) to align the Sanger reads with default settings.

BFAST (v0.7.0a) was used with the default parameters, similarly to the steps described in (1). BFAST is a highly sensitive aligner that generates a large number of alignments of lower mapping quality. During the post-processing step, low quality alignments (MAPQ < 20) were removed to ensure that all pipelines using BFAST alignments finish the indel detection in a reasonable amount of time (within 72 hours on a high-performance computing cluster).

Picard was used to remove duplicate reads from Illumina reads alignment with "VALIDATION_STRINGENCY = LENIENT REMOVE_DUPLICATES = True".

Dindel (v1.01) was used with the default parameters, similarly to the steps described in (1).

GATK (v1.6.5) was used with the options "-T UnifiedGenotyper -glm INDEL" to call indels from Illumina reads alignment.

PRISM (v1.1.5): we set minimum discordant pair number for a cluster to 2. We call an indel when it is supported by at least 5 reads and the best alignment has no more than 2 mismatches.

Section 3: Concordance among indel detection tools

We found only a modest degree of overlap between the different methods: 369,641 indels were detected by all five pipeline combinations used in our analysis (**Figure S3**). The relatively low level of concordance is consistent with the results from literature.

A low concordance among different indel detection methods has been observed in a number of studies, suggesting that indel detection in human populations is likely to be rather incomplete (2). A recent study reported a mere 26.8% agreement among three indel-calling pipelines, which is substantially lower than the concordance for SNP calls (3). These results indicate a potentially high numbers of false positives and/or false negatives. Another recent study found an even lower concordance of only 14.3% among three different indel detection pipelines, and noted different biases among the tools towards detecting short versus long indels (4). The concordance tended to improve, however, after requiring a higher read coverage for one of the pipelines.

Supplementary References

1. Porter, J., Berkhahn, J. and Zhang, L. (2014), *International Conference on Bioinformatics and Computational Biology (BIOCOMP'14)*, Las Vegas, USA, Vol. http://worldcomp-proceedings.com/proc/p2014/BIC.html.
2. Mills, R.E., Pittard, W.S., Mullaney, J.M., Farooq, U., Creasy, T.H., Mahurkar, A.A., Kemeza, D.M., Strassler, D.S., Ponting, C.P., Webber, C. *et al.* (2011) Natural genetic variation caused by small insertions and deletions in the human genome. *Genome research*, **21**, 830-839.
3. O'Rawe, J., Jiang, T., Sun, G., Wu, Y., Wang, W., Hu, J., Bodily, P., Tian, L., Hakonarson, H., Johnson, W.E. *et al.* (2013) Low concordance of multiple variant-calling pipelines: practical implications for exome and genome sequencing. *Genome medicine*, **5**, 28.
4. Ghoneim, D.H., Myers, J.R., Tuttle, E. and Paciorkowski, A.R. (2014) Comparison of insertion/deletion calling algorithms on human next-generation sequencing data. *BMC research notes*, **7**, 864.

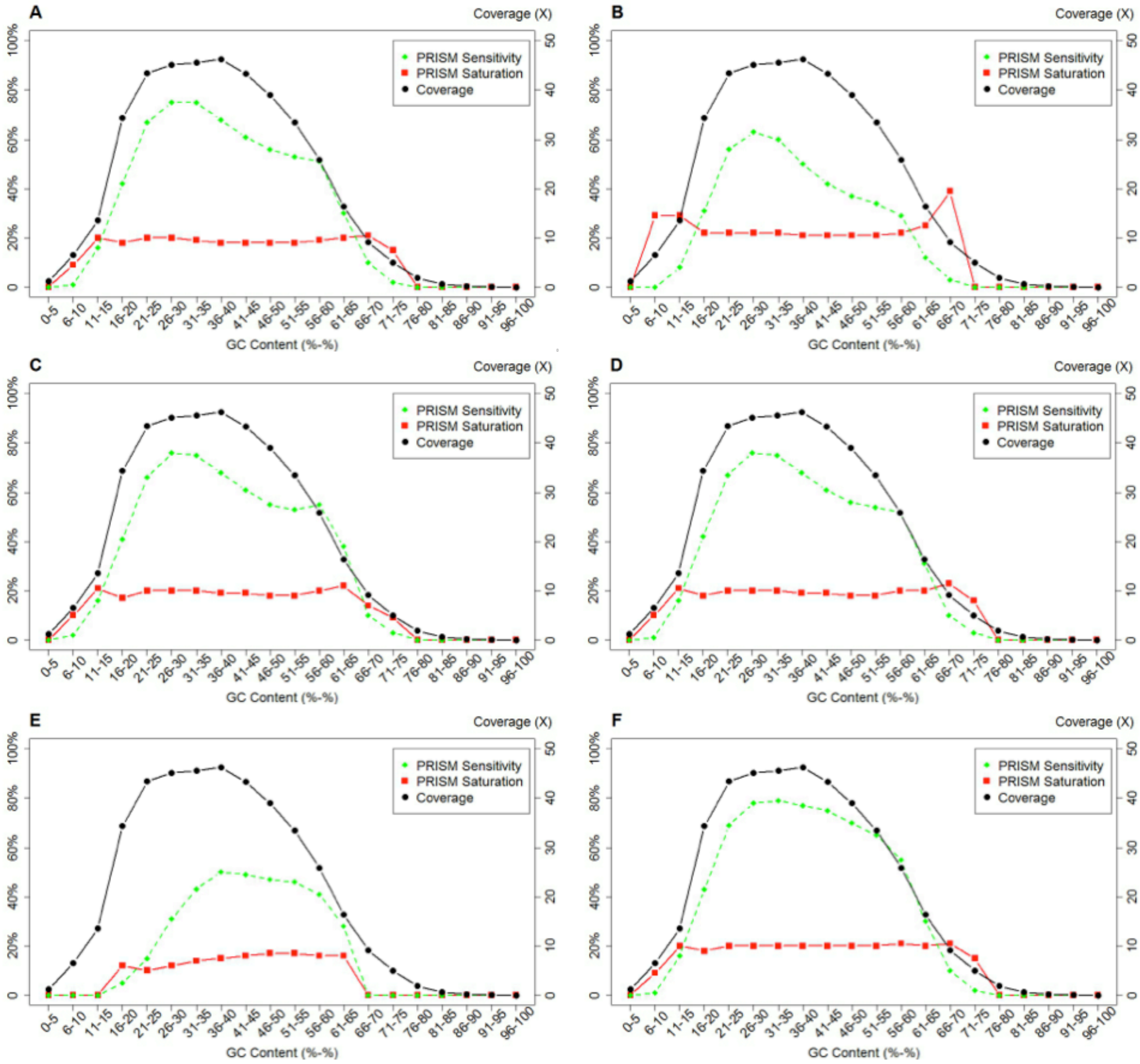

Figure S1. Distribution of PRISM sensitivity, saturation and precision over GC content under different conditions. **A**. Original PRISM indel distribution. **B**. High coverage indels (≥ 10 support reads). **C**. Indels more than 5Mbp away from centromeres and telomeres. **D**. Indels outside UCSC segDup annotations. **E**. Indels within 50bp of Alus. **F.** Indels more than 50bp away from Alu elements. In GC content region of 36%-40%, PRISM sensitivity and saturation decline, although the coverage rises. This situation holds for B-D but not for E. F shows that PRISM detection performance outside of Alu regions is superior to A-E, which together with E indicates that the presence of Alus is the main reason for the loss of detection accuracy.

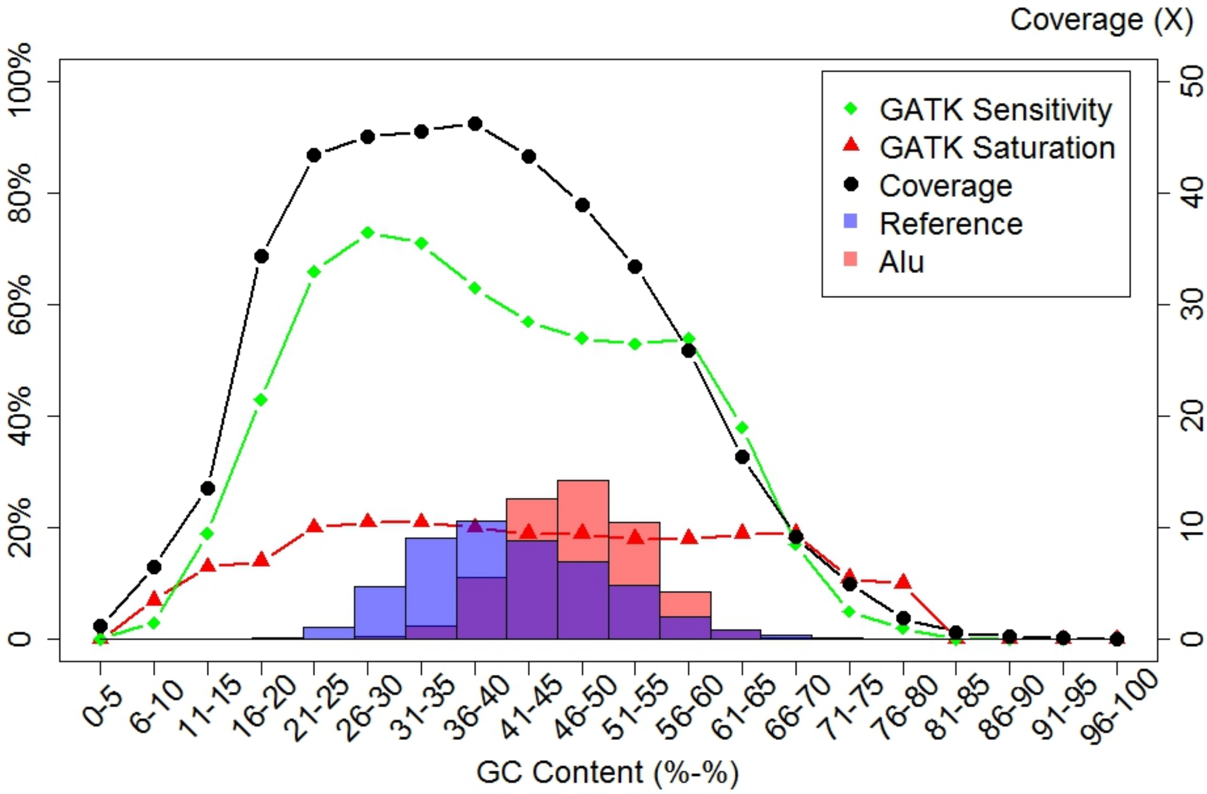

Figure S2. Dependence of coverage and GATK indel-detection metrics on the GC content.
The reference genome was cut into 200bp pieces and binned by GC content. GATK indel detection sensitivity (green curve) and saturation (red curve) are shown for each bin along with the genome coverage (black curve). Also shown is the distribution of the full reference genome across the same GC bins (blue semi-transparent histogram), as well as the distribution of Alu elements (red semi-transparent histogram). The two histograms demonstrate that Alus are generally overrepresented in areas with higher GC content, and also that the noticeable dip in the GATK sensitivity corresponds well with the presence of Alu elements (pink and magenta areas of the Alu histogram).

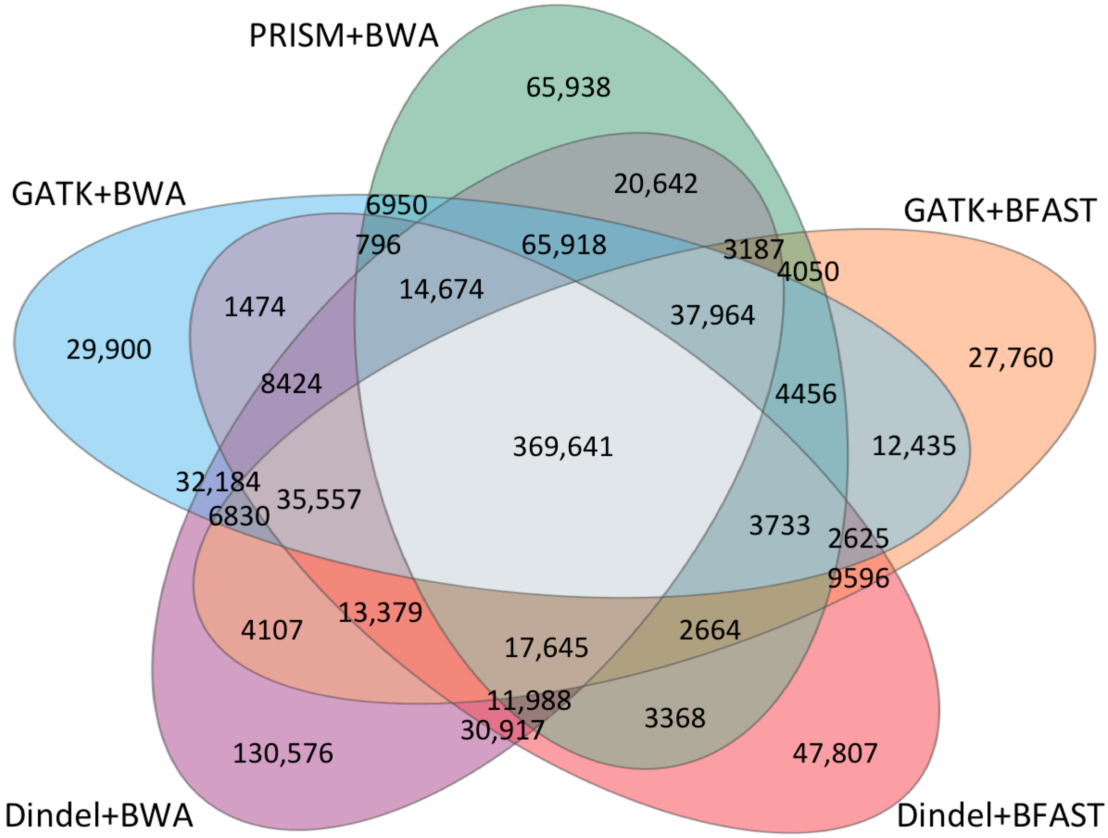

Figure S3. Overlap in indels detected in NA18507 genome by different pipelines. The Venn diagram shows the number of indels detected by different combinations of the read mapper and indel caller. The main results in this study were obtained using the PRISM+BWA pipeline. They were validated using 4 other pipelines, which combined either BWA or BFAST mappers with either GATK or Dindel detection algorithms. A comparable degree of overlap exists between different setups of indel detection tools.

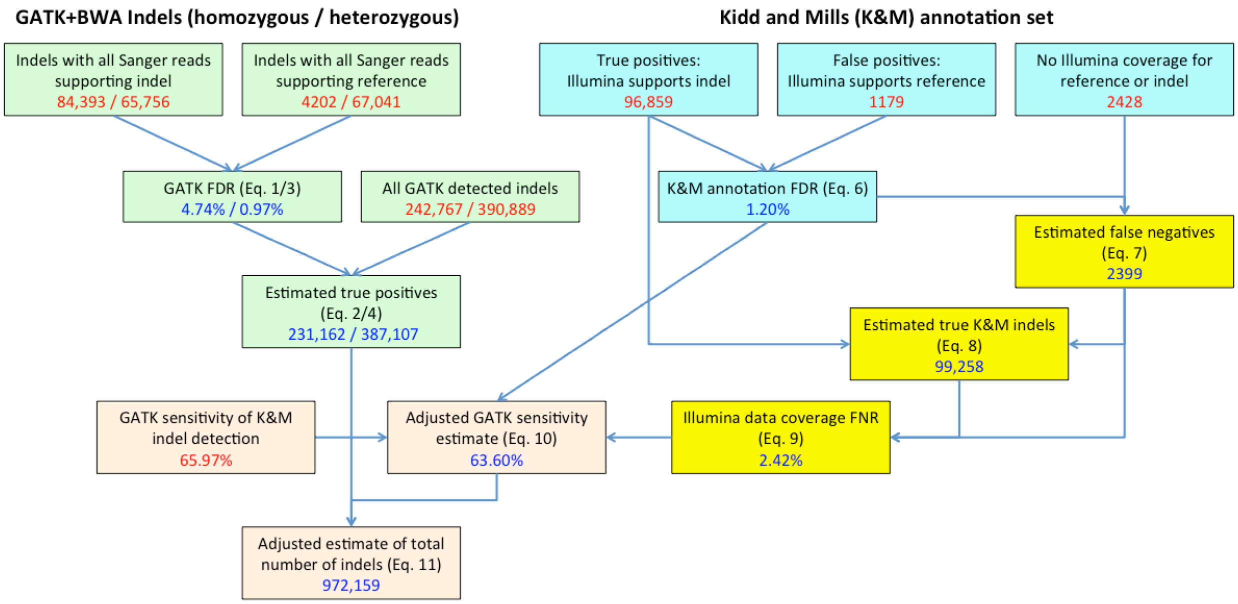

Figure S4. Estimation of the total number of 1-10bp indels in the Yoruban genome NA18507 using GATK in combination with BWA read aligner. The workflow involves the estimation of four sets of values: GATK FDR and the number of true positive indels detected by GATK (green boxes); the reliability of the reference indel annotation combined from Kidd and Mills sets (via false discovery rate, FDR; blue boxes); the incompleteness of the Illumina read coverage of the reference indels (via false negative rate, FNR; yellow boxes); and the computation of the adjusted sensitivity of indel detection in GATK, which is used to estimate the overall number of indels in the genome (orange boxes). Each box shows the initial indel counts or pipeline sensitivity (red numbers) as well as the computed estimates (blue numbers) based on the equations indicated in parentheses. The detailed explanation of the equations and the workflow is presented in Section 1 of this document.

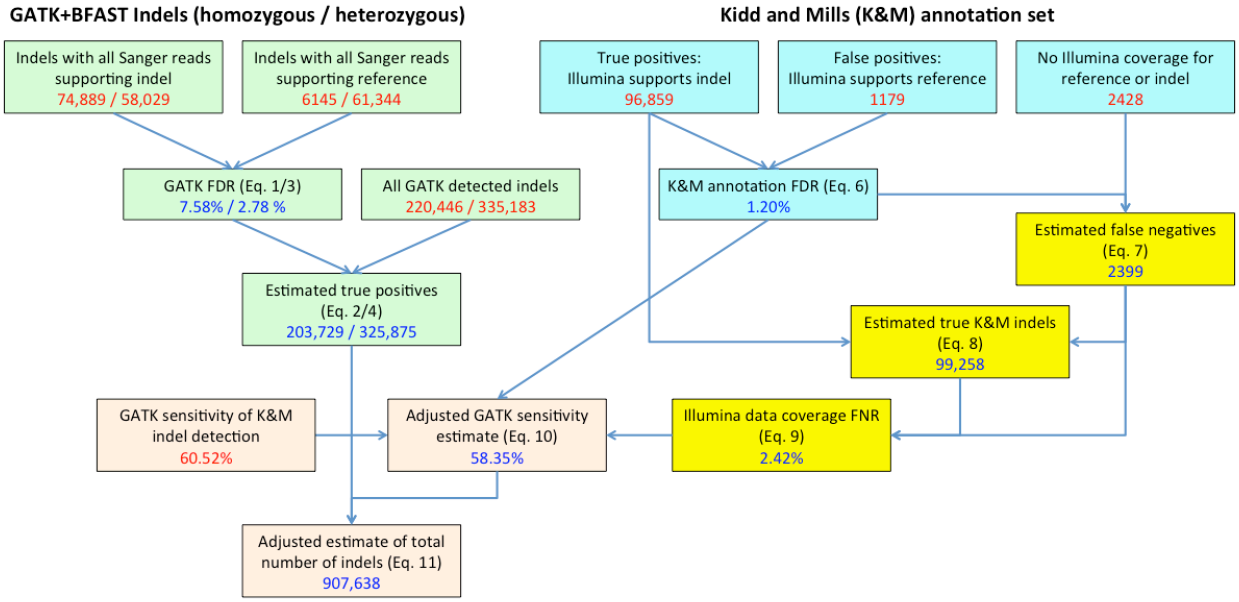

Figure S5. Estimation of the total number of 1-10bp indels in the Yoruban genome NA18507 using GATK in combination with BFAST read aligner.

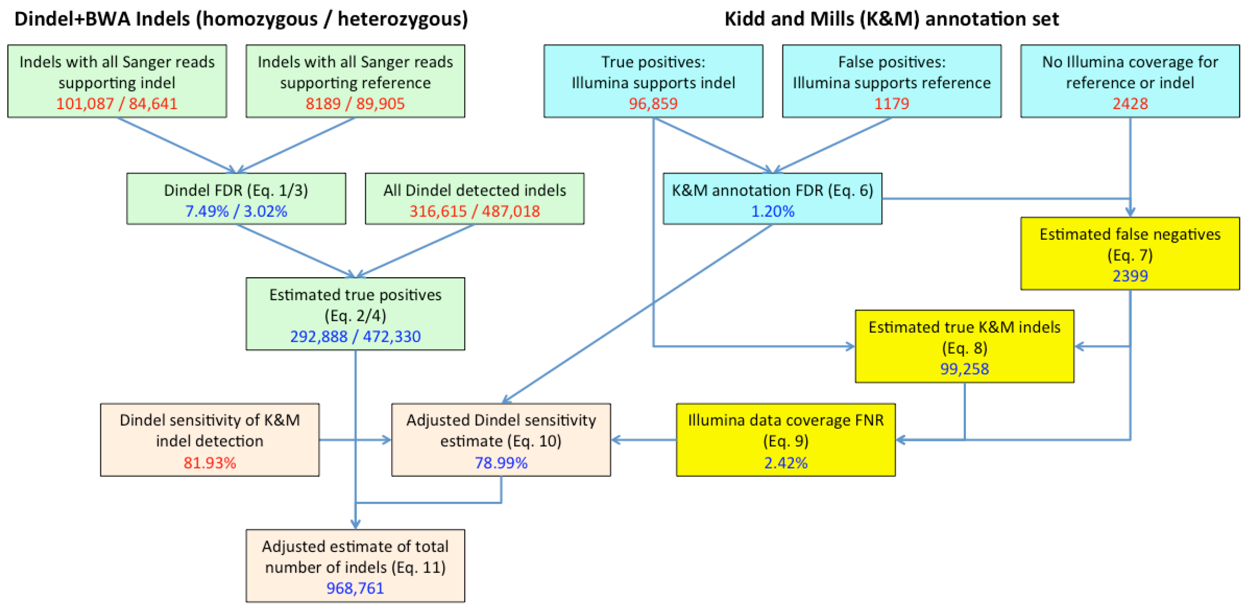

Figure S6. Estimation of the total number of 1-10bp indels in the Yoruban genome NA18507 using Dindel in combination with BWA read aligner.

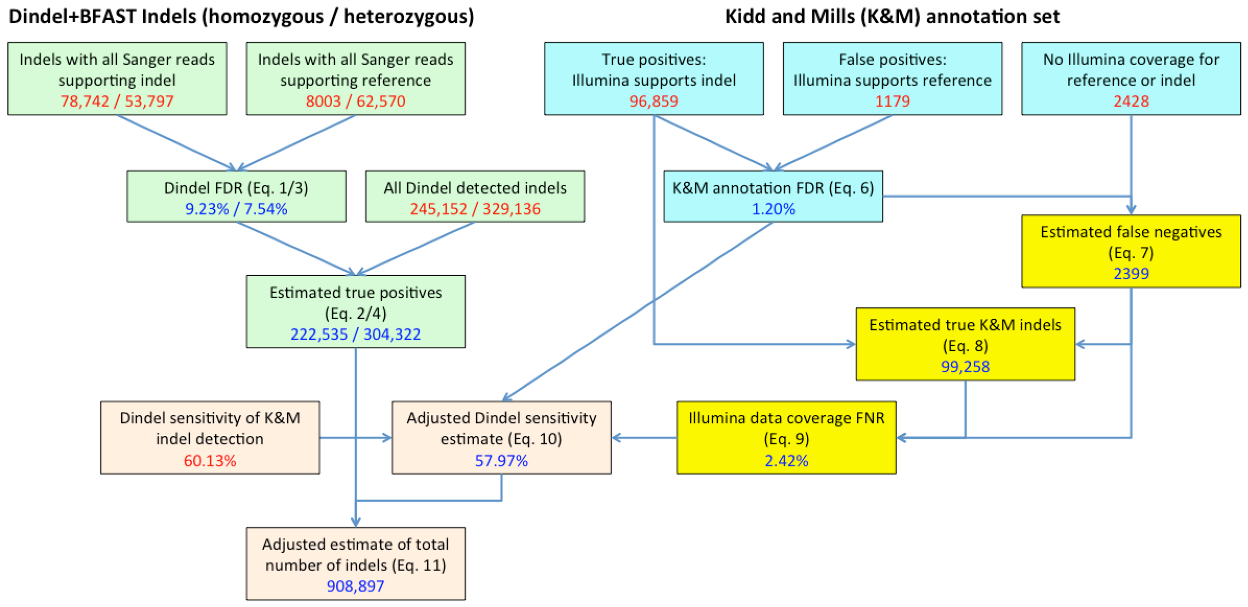

Figure S7. Estimation of the total number of 1-10bp indels in the Yoruban genome NA18507 using Dindel in combination with BFAST read aligner.

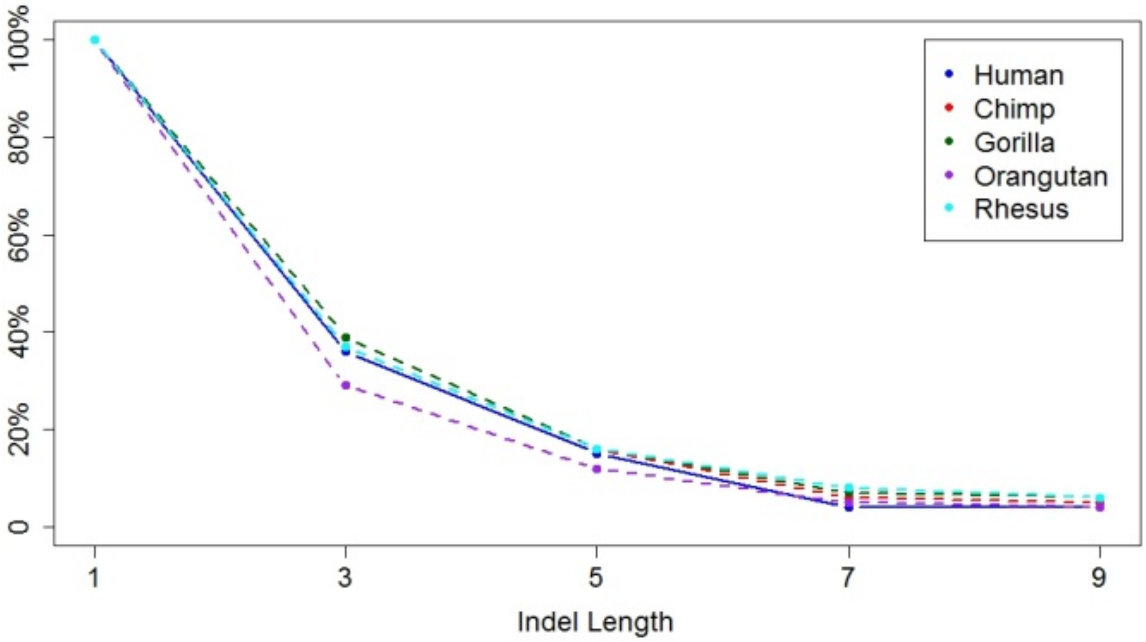

Figure S8. Distribution of indel length in human and four other primates in non-homopolymers. The counts of indels of each length are normalized by the number of 1 bp indels. The fractions of longer indels are very stable across all the five species. Indels of even length are not included due to the existence of dimers whose variation rate can be significantly different from odd length indels.

Table S1. Estimated number of indels in non-homopolymers and short homopolymers (2-10 bp) in the Yoruban genome NA18507. The results from all pipelines are based on the 100bp read set. Indels of length 1-10bp are considered.

Quantity	PRISM +BWA	GATK +BWA	GATK +BFAST	Dindel +BWA	Dindel +BFAST
Total number of detected indels	476,422	462,711	423,224	546,058	423,723
All Sanger reads support variant	111,102	109,489	100,334	125,340	98,523
All Sanger reads support reference	57,120	52,997	53,013	71,075	55,247
Estimated true positives	448,114	440,702	394,147	503,905	385,518
Sensitivity on Kidd ∩ Mills set	72.73%	71.06%	67.09%	85.10%	65.60%
Total estimate	616,133	620,171	587,487	592,138	587,675
Adjusted sensitivity	70.12%	68.51%	64.68%	82.04%	63.25%
Adjusted total estimate	639,077	643,266	609,364	614,189	609,560
